# A survey on *Mycobacterium ulcerans* in Mosquitoes and March flies captured from endemic areas of Northern Queensland, Australia

**DOI:** 10.1371/journal.pntd.0006745

**Published:** 2019-02-21

**Authors:** Avishek Singh, William John Hannan McBride, Brenda Govan, Mark Pearson, Scott A. Ritchie

**Affiliations:** 1 Cairns Clinical School, College of Medicine and Dentistry, James Cook University, Cairns City, Australia; 2 College of Public Health, Medical & Vet Sciences, James Cook University, Townsville, Australia; 3 Australian Institute of Tropical Health & Medicine, James Cook University, Smithfield, Australia; 4 College of Public Health, Medical and Veterinary Sciences, James Cook University, Smithfield, Australia, Australian Institute of Tropical Health and Medicine (AITHM), James Cook University, Smithfield, Australia; University of Melbourne, AUSTRALIA

## Abstract

*Mycobacterium ulcerans* is the causative agent of Buruli ulcer (BU). This nontuberculous mycobacterial infection has been reported in 34 countries worldwide. In Australia, the majority of cases of BU have been recorded in coastal Victoria and the Mossman-Daintree areas of north Queensland. Mosquitoes have been postulated as a vector of *M*. *ulcerans* in Victoria, however the specific mode of transmission of this disease is still far from being well understood. In the current study, we trapped and analysed 16,900 (allocated to 845 pools) mosquitoes and 296 March flies from the endemic areas of north Queensland to examine for the presence of *M*. *ulcerans* DNA by polymerase chain reaction. Seven of 845 pools of mosquitoes were positive on screening using the IS*2404* PCR target (maximum likelihood estimate 0.4/1,000). *M*. *ulcerans* DNA was detected from one pool of mosquitoes from which all three PCR targets: IS*2404*, IS*2606* and the ketoreductase B domain of mycolactone polyketide synthase gene were detected. None of the March fly samples were positive for the presence of *M*. *ulcerans* DNA.

## Author summary

The causative agent of Buruli ulcer is *Mycobacterium ulcerans*. This destructive skin disease is characterized by extensive and painless necrosis of skin and underlying tissues usually on extremities of body due to production of toxin named mycolactone. The disease is prevalent in Africa and coastal Australia. The exact mode of transmission and potential environmental reservoir for the pathogen still remain obscure. Aquatic and biting insects have been identified as potential niche in transmission and maintenance of pathogen in the environment. In this study we screened mosquitoes and march flies captured from endemic areas of northern Queensland for the presence of *M*. *ulcerans* DNA. We found seven pools of mosquito out of 845 pools positive for IS*2404*. In only one of the seven samples were the additional targets IS*2606* and KR detected. None of the March fly samples were positive. The results could indicate a low burden of the bacteria in the environment coinciding with a comparatively low number of human cases of *M*. *ulcerans* infection seen during the trapping period of the study.

## Introduction

Buruli ulcer (BU), also known regionally as Daintree ulcer in north Queensland, Australia or Bairnsdale ulcer in Victoria, Australia, is an emerging disease of skin and underlying tissue, with a potential to lead to permanent disability, particularly if treatment is inadequate or delayed. The causative agent of this disease, *M*. *ulcerans* secretes a polyketide exotoxin, mycolactone, the production of which requires expression of a series of contiguous genes on the large pMUM001 plasmid. This exotoxin is the main virulence determinant of the bacteria [1]. The outbreaks of BU have been consistently linked with wetland or coastal regions [2]. Environmental samples such as water, aquatic plants, soil at endemic areas has been found PCR-positive for *M*. *ulcerans* DNA [3, 4]. Insects such as mosquitoes and aquatic bugs has been proposed as a vital ecological niche for the maintenance of pathogen in environment [5, 6].The detection of *M*. *ulcerans* DNA in insects does not prove their ability to transmit *M*. *ulcerans* but could indicate potential to act as either biological or mechanical vector. A study conducted by Marsollier and his colleagues provided evidence of the presence of *M*. *ulcerans* DNA in the salivary gland of wild caught Naucoridae (aquatic bug). They successfully isolated the pathogen by culture from the salivary glands of aquatic bugs and suggested aquatic insects as having an important ecological niche in the maintenance of the organism in the environment. They were also able to demonstrate transmission to mice in a laboratory environment [6]. Similarly, a study conducted by Wallace *et al*. provided evidence of the ability of mosquitoes to act as a mechanical vector of *M*. *ulcerans* [7]. Studies conducted in endemic areas of Africa suggest that conducting farming activities close to rivers [8] and swimming in rivers located in endemic areas [9] are risk factors for exposure to *M*. *ulcerans*.

In Australia, foci of BU infection have been found in tropical north Queensland [10, 11], the Capricorn Coast region of central Queensland [10], the Northern Territory [12] and temperate coastal Victoria [5]. In Queensland, Australia, cases of Daintree ulcer have been reported primarily in Douglas Shire, exclusively in the vicinity of Wonga, Miallo and Daintree [10, 11]. A few cases has also been reported from Capricorn Coast region of central Queensland [10]. The Douglas Shire covers an area of 2,445 sq. Kms and the total population is around 11,000. A majority of the population (around 70%) reside in Port Douglas and Mossman. Thus, the Daintree ulcer endemic areas in north Queensland is sparsely populated. There has been a significant decrease in human cases of BU in north Queensland, since a large outbreak in 2011–2012, when more than 60 cases were reported. This outbreak occurred after prolonged and heavy rainfall in 2010–2011 [11]. The average reported rate over fifteen years period from 2002–2016 was 0.2 cases/100,000 population per year [13].

Victorian researchers detected the presence of *M*. *ulcerans* DNA in five different species of mosquito during a BU outbreak in an endemic area of Victoria, Australia. They demonstrated the absence of *M*. *ulcerans* in a neighboring area, where BU did not occur [5]. Together, the evidence was proposed to support a link with mosquitoes in the ecology of BU in Victoria [5, 14]. More recently, a small study conducted in the BU endemic region of north Queensland, found that of 35 insect/insects pools, one sample of an individual mosquito and one pool of two mosquitoes were positive for IS*2404*. The IS*2404* positive mosquito pool contained DNA of a closely related *M*. *ulcerans* subspecies that had a low copy number for IS*2606* which does not commonly cause disease in human. The individual mosquito had insufficient DNA for detection of the additional gene targets. The study highlighted a need to examine a larger sample size to gauge the significance of the role of mosquito in ecology of BU in Northern Queensland [15]. An additional suggestion proposed by the local population (including people with a history of BU) was that March flies (Tabanidae) might have a role in transmission. We therefore aimed, in this study to capture and screen mosquitoes and March flies for the presence of *M*. *ulcerans* DNA in the BU endemic area of Northern Queensland.

## Materials and methods

**Fig 1:**
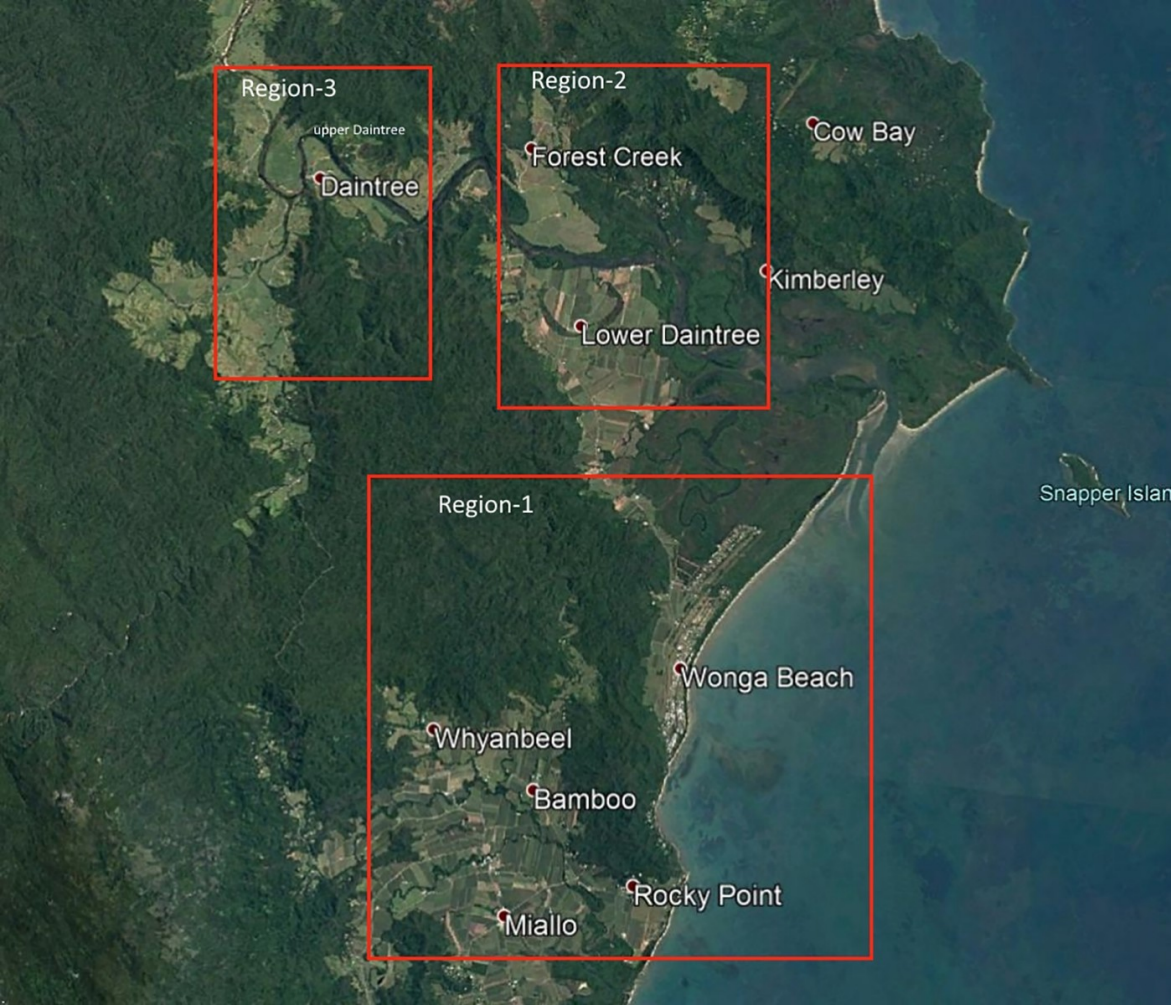
BU endemic areas of Northern Queensland, Australia and Mosquito trapping regions. **This figure was created using base layer obtained from** https://landsatlook.usgs.gov/.

Selection of the study site was based on GIS mapping of human cases of BU in Northern Queensland [16]. We divided the endemic area of northern Queensland into three regions: Region-1: extending from Miallo to lower Daintree including Wonga/Wonga Beach area, Region-2: Forest Creek area and Region-3: Upper Daintree area (Fig 1).

### Trapping of mosquitoes

**Fig 2:**
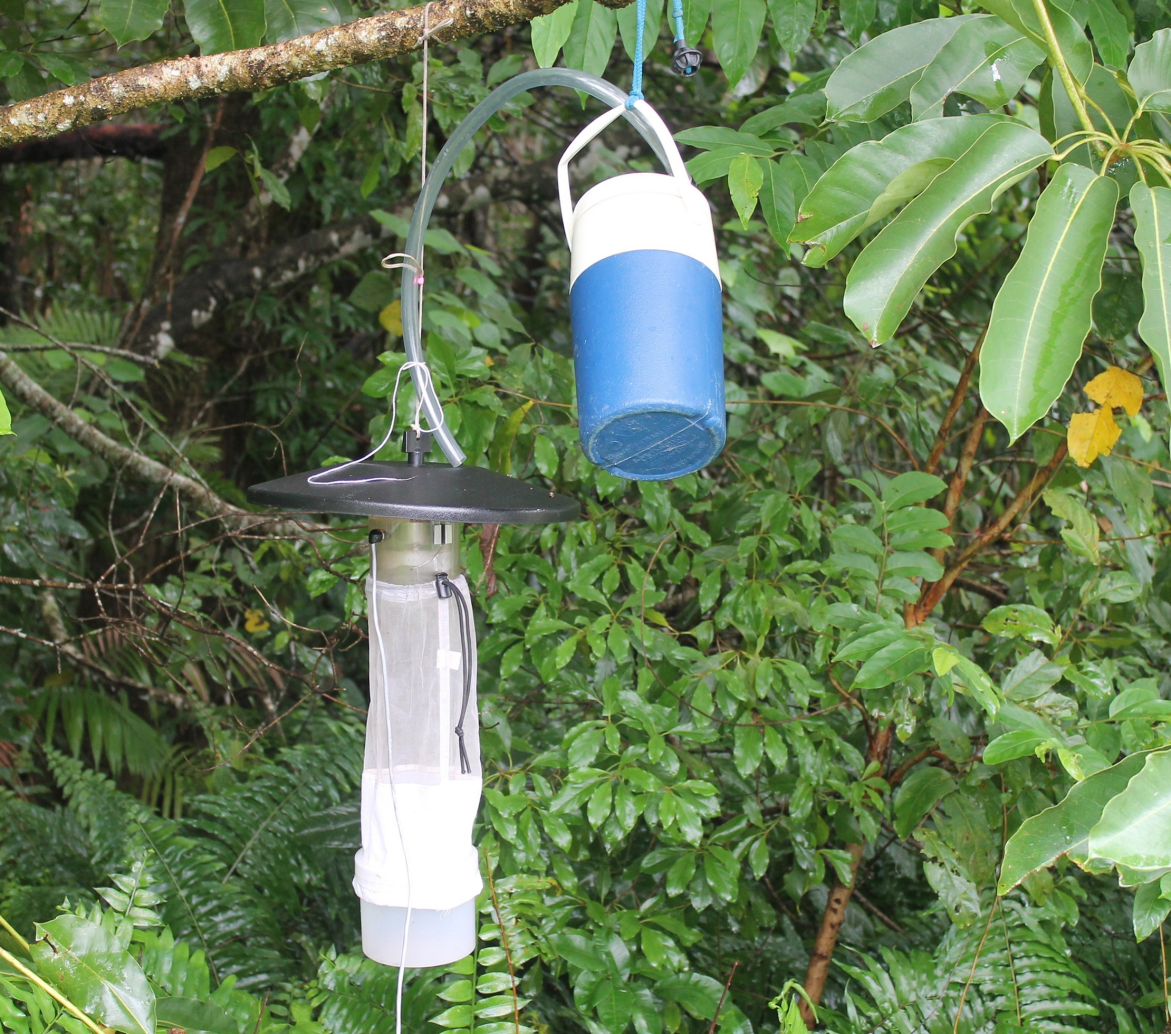
CDC miniature light trap baited with dry ice.

Mosquitoes were captured using a model 512 “CDC miniature light trap” (John W. Hock Company, Gainesville Florida USA) baited with 1 kg of dry ice as the source of CO_2_. This trap is the most reliable, efficient and portable device for trapping mosquitoes and sand flies [17]. This trap consists of an electric light and fan just over the collection container and is operated by a 12V battery. A two liter insulated container was used to hold dry ice and a pipe was attached to release CO_2_ over the trap to attract mosquitoes (Fig 2). Thirty overnight trapping sessions were conducted starting from September 2016 through to February 2018, with at least 4 CDC traps placed within a 1 kilometer radius of each-other. Of the 30 trapping sessions, 14 were conducted at eight different sites within region-1, nine at six different sites within region-2 and seven at five different sites of region-3 (Fig 1). Traps were placed at different sites after obtaining permission to access properties from the owners and selection of sites were based on history of BU cases in humans in nearby households. Geographical Information System (GIS) coordinates of each trap was recorded. On each occasion, traps were set before dusk and checked for mosquitoes after dawn the next morning. After each occasion of trapping, catches were transported to the Mosquito Research Facility, Australian Institute of Tropical Health and Medicine (AITHM), James Cook University, Cairns, Australia where they were counted, sorted and pooled by genus, with each pool containing ≤ 20 mosquitoes of same genus and collected from the same site. The key of Russell was used to identify the genus of mosquitoes trapped [18].

### Trapping of march flies

Several attempts were made to trap march flies from endemic areas with an investigator wearing dark clothes to attract them, or with the use of an insect net sprayed with insecticide. These attempts occurred from February 2016 through September 2016. The yield from these attempts were very low. A request was made to residents of region-1 through the local State School to collect march flies. This effort was successful and large numbers of March flies of genus *Tabanus* were collected by the local community. The addresses of properties from which March flies were collected were recorded. Sampling of March flies was restricted to region-1.

### Molecular analyses

The molecular analyses were performed using the protocol available on given link: dx.doi.org/10.17504/protocols.io.vqbe5sn.

### Screening of mosquitoes and march flies for MU DNA by PCR

DNA was extracted from each pools of ≤ 20 mosquitoes of the same genus by using the FastPrep Instrument (MP Biomedicals, Solon, OH, USA) as per manufacturer’s instruction with FastDNA Kit (MP Biomedicals). Using the same instrument, DNA from individual March fly was extracted with FastDNA Spin Kit (MP Biomedicals). One sterile water sample in each batch of extractions was used as a negative control to identify the possible contamination during the process of extraction of DNA. Extracted DNA was stored at -20 ^o^C. The extracted DNA samples were screened for the presence of *M*. *ulcerans* DNA by using a semi-quantitative real-time PCR adapted from a method for the detection of *M*. *ulcerans* DNA from environmental samples [19]. To rule-out the possibility of contamination, three negative controls (double deionized water, MilliQ) and three positive controls (purified *M*. *ulcerans* DNA obtained from Victorian Infectious Disease Reference Laboratory) were used during qPCR assay run. All of the extracted DNA samples were initially screened for the *M*. *ulcerans* insertion sequence (IS) element IS*2404*. Samples positive for IS*2404* were re-analyzed by a second real-time PCR for the detection of two additional regions in the genome of *M*. *ulcerans*: IS*2606* and ketoreductase B domain (KR). This screening process has been validated by Fyfe *et al*. to differentiate *M*. *ulcerans* from other mycolactone producing mycobacteria (MPM) [19]. They suggested that the difference in real-time PCR cycle thresholds (Ct) between IS*2606* and IS*2404* (ΔCt [IS*2606* –IS*2404*]) allows for the differentiation of *M*. *ulcerans* strains commonly causing disease in human from other MPM (which are also considered members of the species *M*. *ulcerans*) that contain IS*2404* but which have fewer copy numbers of IS2*606*. Samples containing all three independent DNA sequences and with expected Ct values were considered positive for *M*. *ulcerans* DNA. The software recommended by Centers of Disease Control and Prevention (Atlanta, GA, USA) was used to calculate the maximum likelihood estimate (MLE) per 1,000 mosquitoes tested (bias corrected MLE) [20].

### Accession numbers

The Genebank accession number of nucleotide sequence on *M*. *ulcerans* gene IS*2404*, IS*2606* and KR have been allocated as BX649209, BX649209 and BX649209 respectively.

## Results

### Screening of mosquitoes

**Fig 3:**
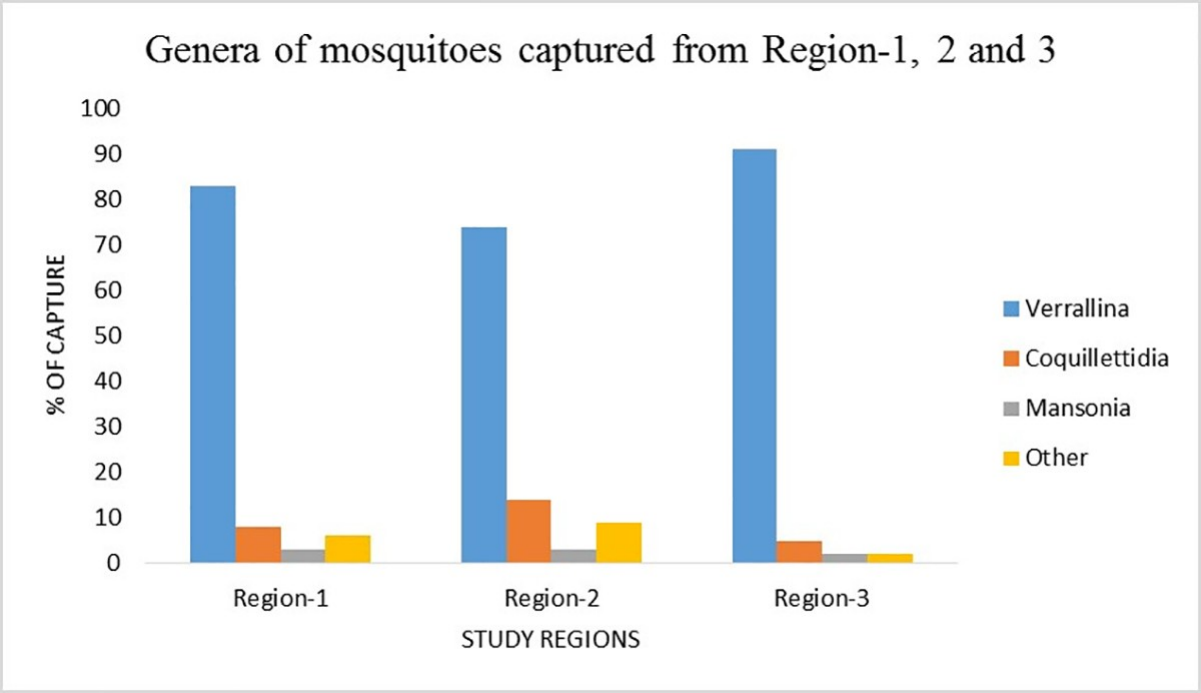
Genera of mosquitoes captured from three different regions: Region-1 comprising 83% of *Verrallina sp*., 8% of *Coquillettidia sp*., 3% of *Mansonia sp*. and 6% of others; Region-2 comprising 74% of *Verrallina sp*., 14% of *Coquillettidia sp*., 3% of *Mansonia sp*. and 9% of others and Region-3 comprising 91% of *Verrallina sp*., 5% of *Coquillettidia sp*., 2% of *Mansonia sp*. and 2% of others of total catches.

A total of 16,900 mosquitoes were captured over the course of the study from 30 occasions of trapping at three different regions of northern Queensland. Total mosquitoes captured from region-1, region-2 and region-3 were 7880, 5100, and 3920, respectively. The majority of captured mosquitos belonged to the *Verrallina* genus (specifically *Verrallina lineata*) 82%, followed by *Coquillettidia* (9%) and *Mansonia* (3%). The remaining 6% consisting seven other genera that were classified as “other” for screening. See Fig 3 below.

Of a total of 16,900 mosquitoes screened (845 pools), seven pools were positive for IS*2404*. Three of those seven pools were *Verrallina sp*. from region-1, two pools were *Coquillettidia sp*. one each from capture region-1 and 3 and the remaining two pools were *Mansonia sp*. from region-1. Of the seven pools positive for IS*2404*, two pools had a high cycle threshold (Ct) values for IS2404 and did not contain sufficient amount of DNA to detect IS*2606* and KR. IS*2606* was not detected from four pools, despite of having desired Ct values for IS*2404*. All three targets were detected from remaining pool (Table 1). Thirty pools of mosquitoes which were negative for IS*2404* were tested for IS*2606* and KR. None of them were positive for these probes signifying the dependent nature of existence of IS *2606* and KR with IS*2404*. Similar findings were reported during the Victorian outbreak [5]. The bias corrected MLE value for all mosquitoes collected from study site (region-1, region-2 and region-3) was 0.06 *M*. *ulcerans* PCR-positive mosquitoes per 1,000 tested (95% confidence interval, 0.00–0.29). Only Region-1 had *M*. *ulcerans* PCR-positive mosquitoes and calculated MLE value was 0.13 (95% confidence interval,0.01–0.61)/1,000 mosquitoes tested.

**Table 1:** Ct values on qPCR analysisScreening of march flies.

Samples	Species	Location and collection	qPCR analysis			
			IS*2404*	IS*2606*	IS*2404-*IS*2606*	KR
Mosquito Pool-1	*Verrallina sp*.	Region-1; Feb 2017	31.1	32.9	1.8	27.6
Mosquito Pool-2	*Verrallina sp*.	Region-1; March 2017	31.3	ND	ND	ND
Mosquito Pool-3	*Verrallina sp*.	Region-1;Aug 2017	36.1	ND	ND	ND
Mosquito Pool-4	*Coquillettidia sp*	Region-1; Feb 2017	31.2	ND	ND	ND
Mosquito Pool-5	*Coquillettidia sp*	Region-3; Sep 2017	30.4	ND	ND	ND
Mosquito Pool-6	*Mansonia sp*.	Region-1; Feb 2017	32.6	ND	ND	ND
Mosquito Pool-7	*Mansonia sp*.	Region-1; Aug 2017	38.2	ND	ND	ND

DNA extracts of 296 March flies were screened for IS*2404*. None of the samples were positive for this probe. Twenty-four randomly selected IS*2404* negative samples were tested for IS*2606* and KR and none were positive.

## Discussion

Mosquitoes serve as important biological vectors for a variety of pathogens. The movement of pathogens from the gastro-intestinal tract after ingestion to the salivary glands for subsequent transmission is well documented for many diseases. However, this phenomenon has not been demonstrated for *M*. *ulcerans*. A study conducted by Wallace and colleagues (2010) provided evidence on the maintenance of *M*. *ulcerans* throughout larval development without further passage of the organisms into pupa or adult mosquitoes [21]. They concluded that mosquitoes were an unlikely biological vector of *M*. *ulcerans*. Wallace *et al* (2017) subsequently provided evidence of mechanical transmission of *M*. *ulcerans* via anthropogenic skin puncture or mosquito bites [7].

For mechanical transmission, insect vectors such as mosquitoes must acquire the pathogen either from the environment or an infected host. For this to occur efficiently, the organism must be abundantly present in the environment. A survey in Victoria, Australia has confirmed a strong correlation between mosquitoes found to test positive for carrying *M*. *ulcerans* DNA and the number of human cases of BU occurring [5, 22]. The group found a significantly higher number of mosquitoes screened positive for *M*. *ulcerans* DNA during an intense outbreak of BU in endemic areas, in comparison to areas with a lower incidence of human cases.

The number of human cases of BU has decreased in Northern Queensland, Australia since the largest recorded outbreak in 2011 (> 60 cases). The majority of the cases during the 2011 outbreak were from Wonga and the Wonga beach area, referred as region-1 in the study by Steffen and Freeborn (2018) [23]. Out of 394 pools collected in region 1, six pools were positive for IS*2404* DNA in this study. Interestingly, three pools mosquitoes of these positive pools were trapped in the backyard of a property in Wonga Beach area (region-1) where two human cases of BU were confirmed in 2017. All other pools of mosquitoes and march flies collected from that properties negative for *M*. *ulcerans* DNA.

As shown in the result, seven pools of mosquitoes were positive for IS*2404*. However, all three targets with expected Ct value were detected from only one of these seven pools. Samples that were positive for only IS*2404* were not considered further.

In north Queensland, the Daintree River arises in mountainous rainforest region around the town of Mossman and flows into the sea at Cape Tribulation. The wet season starts normally from November/December and continues up to April, and the dry season starts from May and continues up to October/November. Outbreaks of human cases of BU in north Queensland have been linked with heavy rainfall and flooding. This survey was conducted from September 2016 through to February 2018, when dryer environmental conditions prevailed. Out of seven *M*. *ulcerans* DNA positive pools of mosquitoes, five were collected in wet season and two were collected in dry season. A majority of cases of Daintree ulcer are reported after rainy season ends [13]. The estimated mean incubation period of Daintree ulcer is 4.5 months [24]. Thus, it is more likely that the transmission occurs in the wet season which justifies the detection of *M*. *ulcerans* DNA from the pools of mosquitoes that were captured in wet season in this study.

In a separate study conducted in North Queensland, Australia, one sample of a single mosquito and one pool of two mosquitoes was found positive for IS*2404*.[15]. However, it must be noted that this study was conducted soon after 2011 which raises the possibility that sampling should occur as close as possible in time to when transmission is thought to be occurring.

*M*. *ulcerans* is an environmental pathogen and detection of *M*. *ulcerans* DNA positive mosquitoes may only be an indicator for the presence of the organism in the environment. A significant decrease in human cases of BU in Northern Queensland in recent years could be due to a lower load of bacteria in the environment. This may explain the low detection of *M*. *ulcerans* DNA positive mosquitoes and March fly populations in the study sites. However, the detection of *M*. *ulcerans* DNA even in a single pool of mosquitoes from the endemic areas of Northern Queensland is significant, as it corroborates findings in Victoria where five different species of mosquitoes captured from BU-endemic regions during human outbreaks were positive for *M*. *ulcerans*.

Our detection of *M*. *ulcerans* DNA in mosquitoes in Northern Queensland does support the earlier report from Victoria in Australia [5]. The Victorian study provides evidence for high detection rates of *M*. *ulcerans* positive mosquitoes if captured during peak times of outbreaks. Our study found that it is less likely to find *M*. *ulcerans* positive mosquitoes if they are trapped from areas where human incidence of BU is currently low. We hypothesize that mosquitoes and perhaps other biting insects, such as March flies may have a significant role in the ecology and transmission of *M*. *ulcerans* in endemic areas during outbreaks and that the level of detection of *M*. *ulcerans* positive mosquitoes in the environment could be an indicator for disease outbreaks.

## Conclusions

Our study confirms the presence of *M*. *ulcerans* DNA in the mosquitoes samples captured from the BU-endemic regions of North Queensland, Australia. Lower detection of *M*. *ulcerans* positive mosquitoes in BU-endemic areas in North Queensland may partially explain low endemicity of the disease.
